# The psychedelic (−)-2,5-dimethoxy-4-iodoamphetamine [(−)-DOI] demonstrates efficacy in reducing cocaine reward and motivation in male rats

**DOI:** 10.1007/s00213-025-06765-3

**Published:** 2025-03-04

**Authors:** Leah M. Salinsky, Christina R. Merritt, Erik J. Garcia, Robert G. Fox, Joshua C. Zamora, Noelle C. Anastasio, Kathryn A. Cunningham

**Affiliations:** The Center for Addiction Sciences and Therapeutics and Department of Pharmacology and Toxicology, John Sealy School of Medicine, University of Texas Medical Branch, Galveston, TX USA

**Keywords:** 5-HT_2A_R, Behavioral economics, Cocaine, (−)-2,5-dimethoxy-4-iodoamphetamine, Psychedelics, Self-administration, Serotonin

## Abstract

**Rationale and objectives:**

Overdose fatalities involving cocaine continue to rise with over 5.3 million cocaine users reported in the United States in 2022. The abuse liability of cocaine is reliant upon inhibition of dopamine (**DA**) reuptake and consequent increase in DA efflux in meso-corticolimbic circuitry that controls reward and motivation. Cocaine also increases serotonin (**5-HT**) efflux which is integral in cocaine abuse. The 5-HT_2A_ receptor (**5-HT**_**2A**_**R**) is a key regulator of meso-corticolimbic DA release and controls cellular mechanisms underlying cocaine effects. 5-HT_2A_R actions contribute importantly to psychedelic mechanisms of action, and the efficacy of these compounds in limiting cocaine intake is unknown. The present studies evaluated the efficacy of acute administration of a psychedelic to reduce cocaine intake using standard and advanced preclinical models of drug self-administration.

**Methods:**

Both a standard fixed ratio (**FR**) schedule and behavioral economics threshold procedure of cocaine intravenous self-administration were employed to evaluate the efficacy of the psychedelic 5-HT_2A_R agonist (−)-2,5-dimethoxy-4-iodoamphetamine [**(−)-DOI**] to decrease cocaine intake and motivation for cocaine in male rats. The 5-HT_2A_R-selective antagonist M100907 was utilized to explore the role of 5-HT_2A_R in the effects of (−)-DOI on cocaine intake.

**Results:**

We found that (−)-DOI dose-dependently reduced intake on the FR5 schedule of cocaine IVSA and left shifted the demand curve to evoke greater sensitivity to price increases in the behavioral economics paradigm. Pretreatment with M100907 abated the efficacy of (−)-DOI on cocaine intake in both paradigms.

**Conclusion:**

(−)-DOI ‘devalued’ cocaine reward and motivation to take cocaine in a 5-HT_2A_R-dependent manner. As serotonergic psychedelics emerge as therapeutic candidates, investigations of 5-HT_2A_R-acting psychedelics in preclinical analyses of cocaine intake and relapse vulnerability during abstinence will be valuable as prelude to future clinical trials.

## Introduction

The U.S. remains in the grip of the overdose crisis with personal and societal costs estimated at $1.02 trillion in 2017 (Florence et al. 2021). While opioid-involved fatalities decreased in 2023, those involving cocaine continue to rise (Centers for Disease Control and Prevention 2024). The abuse liability of cocaine is reliant upon inhibition of dopamine (**DA**) transporters (Ritz et al. 1987) and the resulting increase in DA efflux (Nicolaysen et al. 1988), prolonging stimulation of DA receptors within meso-corticolimbic circuitry that controls reward and motivation (for reviews) (Bobadilla et al. 2017; Koob and Volkow 2016; Volkow et al. 2007). Cocaine also inhibits serotonin (**5-HT**) reuptake (Ritz et al. 1987) which is integral in CUD processes (for reviews) (Cunningham et al. 2020; Howell and Cunningham 2015; Kirby et al. 2011). Of the 14 5-HT-responsive receptors, the 5-HT_2A_ receptor (**5-HT**_**2A**_**R**) is a key regulator of meso-corticolimbic DA release (Pehek et al. 2006) and controls cellular mechanisms underlying cocaine effects (Franklin and Carrasco 2015; Huang et al. 2009; Wright et al. 2017). The selective 5-HT_2A_R antagonist M100907 (5-HT_2A_R nM affinity; 100 × selectivity vs. the homologous 5-HT_2B_R, 5-HT_2C_R and other GPCRs) (Barnes et al. 2021; Kehne et al. 1996) suppresses the efficacy of experimenter-delivered cocaine to evoke hyperactivity (Fletcher et al. 2002b) and to reinstate cocaine intravenous self-administration (**IVSA**) after extinction (Fletcher et al. 2002b; Nic Dhonnchadha et al. 2009). M100907 dose-dependently also blunts cocaine-seeking driven by discrete cocaine-associated cues in rats (Anastasio et al. 2020; Fletcher et al. 2002a; Pockros et al. 2011; Sholler et al. 2019) and primates (Murnane et al. 2013). However, M100907 does not alter ongoing cocaine IVSA in rodents (Fletcher et al. 2002a; Nic Dhonnchadha et al. 2009; Pockros et al. 2011) or primates (Murnane et al. 2013) at doses lacking effects on ancillary behavioral measures (Cunningham et al. 2013; Fletcher et al. 2002b; McMahon and Cunningham 2001).

Compounds acting as 5-HT_2A_R agonists have not been profiled in cocaine self-administration models, likely because activation of 5-HT_2A_R signaling mediates the psychedelic effects of the classical hallucinogens *d*-lysergic acid diethylamide (Barrett et al. 2018; Kraehenmann et al. 2017; Preller et al. 2017) and psilocybin (Kometer et al. 2012, 2013; Quednow et al. 2012). In rodents, the investigational hallucinogen 2,5-dimethoxy-4-iodoamphetamine (**DOI**) decreased intake of the opioid agonists heroin (Bonilla et al. 2024) and fentanyl (Martin et al. 2021), outcomes blocked by M100907 pretreatment. Based upon the wealth of knowledge depicting the role of 5-HT in CUD processes (for reviews) (Cunningham and Anastasio 2014; Cunningham et al. 2020; Howell and Cunningham 2015), we explored the dose-dependent effects of DOI in adult male rats trained on cocaine IVSA.

DOI is a racemic mixture of (+)- and (−)-isomers, and the present studies utilized (−)-DOI given its higher binding density to the 5-HT_2A_R in rat cortex relative to (+)-DOI (Nazarali et al. 1989). The standard fixed ratio (**FR**) and behavioral economics schedule were employed to illuminate specific reinforcing and motivational aspects of cocaine intake altered by (−)-DOI treatment. The behavioral economics procedure interrogates drug reward value and the motivation to use drug by estimating drug demand as a function of its ‘price’ (responses required/mg) (Hursh and Silberberg 2008) and across a range of doses. Notably, rodents more resilient to price changes in a cocaine demand schedule are likely to continue self-administration despite aversive consequences (i.e., foot shock), display higher response rates during extinction training, and are more sensitive to cue-induced and cocaine-primed reinstatement (Bentzley et al. 2014). Further, self-reported cocaine demand in humans is associated with the quantity, frequency, and severity of use (Strickland et al. 2016). Thus, the conducted experiments provide important translational value with regard to the efficacy of a psychedelic to alter facets of cocaine reward.

## Materials and methods

***Animals.*** Male Sprague–Dawley rats (n = 44; Harlan, Inc., Indianapolis, IN, USA) weighed 225–250 g upon arrival, acclimated for seven days in a colony room with controlled temperature (21–23 °C) and humidity (45–50%) on a 12-h light dark cycle (lights on 0600–1800 h). Rats were pair-housed and weighed and handled daily by the experimenter throughout the study. Rats were freely fed with food and water available throughout all phases of the studies, except during operant training sessions. All experiments were carried out in accordance with the *National Institutes of Health Guide for the Care and Use of Laboratory Animals (2011)* and with approval from the University of Texas Medical Branch Institutional Animal Care and Use Committee.

***Drugs.*** (−)-Cocaine hydrochloride (National Institute on Drug Abuse Drug Supply Program, Bethesda, MD) was dissolved in 0.9% NaCl. (−)-DOI hydrochloride (Sigma, Research Triangle Park, NC, USA) was dissolved in 0.9% NaCl. M100907 [(R)-(2,3-dimethoxyphenyl)-[1-[2-(4-fluorophenyl)ethyl]piperidin-4-yl]methanol] (National Institute on Drug Abuse, Bethesda, MD) was dissolved in 1% Tween-80 in 0.9% NaCl. The term vehicle (**VEH**) is used consistently throughout the text to represent the control group for treatment comparisons.

***Surgical Implantation of Intravenous Catheters.*** Rats were implanted with intravenous catheters with back mounts under anesthesia with a cocktail containing 8.6 mg/kg of xylazine, 1.5 mg/kg of acepromazine, and 43 mg/kg of ketamine in saline and allowed to recover for seven days prior to initiation of IVSA training (Anastasio et al. 2014a, 2014b; Cunningham et al. 2013; Merritt et al. 2023; Neelakantan et al. 2017; Sholler et al. 2019). Catheter patency was evaluated and maintained by daily flushes with a solution of 0.1 ml saline containing heparin sodium (10 U/ml; American Pharmaceutical Partners, East Schaumburg, IL), streptokinase (0.67 mg/ml; Sigma Chemical), and ticarcillin disodium (66.67 mg/ml; Research Products International, Mt. Prospect, IL) immediately following daily cocaine IVSA sessions.

***Apparatus.*** Standard operant conditioning chambers (Med-Associates, Inc., St. Albans, VT) housed in ventilated, sound-attenuating cubicles with fans (Med-Associates, Inc.) were utilized for cocaine IVSA training. Each chamber was equipped with a pellet receptacle flanked by two retractable response levers, a stimulus light above each response lever, and a house light opposite the levers. Cocaine infusions were delivered via syringes attached to infusion pumps (Med-Associates, Inc.) located outside the cubicles. The infusion pumps were connected to swivels (Instech, Plymouth Meeting, PA) fastened to the catheters via polyethylene 20 tubing encased inside a metal spring leash (Plastics One, Roanoke, VA). The right or left lever was randomly assigned as the designated active lever for subjects in this study.

**Impact of (−)-DOI on Cocaine IVSA.** Freely fed rats (n = 16) were trained to lever press for cocaine infusions (0.25 mg/kg/inf) using established methods (Anastasio et al. 2014a, 2014b; Cunningham et al. 2013; Merritt et al. 2023; Sholler et al. 2019). The dose of cocaine utilized (0.25 mg/kg/inf) is well established in the literature to support reliable IVSA (Burbassi and Cervo 2008; Carroll and Lac 1997; Cunningham et al. 2011; Fletcher et al. 2002a; Grottick et al. 2000). This dose falls near the peak of the cocaine IVSA dose–response curve in Sprague–Dawley rats (Barrett et al. 2004; Edwards et al. 2007; Mierzejewski et al. 2003), thus providing an ample window to observe potential suppressive effects of pharmacological intervention with (−)-DOI. Cocaine IVSA training consisted of daily 60-min sessions during which rats were trained to press the active lever to obtain an infusion on a FR schedule of reinforcement. Rats were initially trained on an FR1 schedule until they met a criterion with < 10% variation in the number of infusions received over three consecutive sessions. After demonstration of stable cocaine IVSA on an FR1 schedule, rats progressed to an FR3 schedule, and then on an FR5 schedule once the same criterion levels were met. Schedule completions on the active lever resulted in delivery of a cocaine infusion over a 6-s period paired simultaneously with illumination of the house and stimulus lights and activation of the infusion pump; inactive lever presses produced no scheduled consequences. Following reinforcer delivery, the stimulus light and infusion pump were inactivated for an additional 20-s to indicate a timeout period during which lever presses had no scheduled consequences while the house light remained illuminated. Once stable cocaine IVSA on an FR5 schedule was achieved (< 10% variation in the number of infusions received for three consecutive sessions), the efficacy of (−)-DOI to alter cocaine intake was assessed. Doses and pretreatment times for (−)-DOI were selected from the literature (Canal et al. 2010; Filip et al. 2004; Krebs-Thomson et al. 1998; Wischhof and Koch 2012). Pretreatment times for M100907 (30 min) (Cunningham et al. 2013; Nic Dhonnchadha et al. 2009; Sholler et al. 2019) and (−)-DOI (20 min) (Wischhof and Koch 2012) were based on previous literature. The dose of M100907 was selected from the literature based on the ability to significantly blunt the 5-HT_2A_R-mediated (−)-DOI-induced head twitch response (Canal et al. 2013; Fantegrossi et al. 2010). Tests were administered in a pseudorandomized order with a minimum of two intervening sessions of cocaine IVSA followed by a VEH test to assure stability of baseline responding. Pretreatment with the 5-HT_2A_R antagonist M100907 was employed to confirm that the effects of (−)-DOI on cocaine IVSA were 5-HT_2A_R mediated. (−)-DOI was administered subcutaneously (s.c.) and M100907 was administered intraperitoneally (i.p.); all injections were given at 1 ml/kg volume. Six rats failed to meet acquisition/maintenance criteria or lost catheter patency and were excluded from subsequent analyses. Rats were tested in a pseudorandomized order with a dose–response curve of (−)-DOI (0.03, 0.1, 0.3 mg/kg), followed by combination tests of M100907 (0.01 mg/kg) + VEH and M100907 (0.01 mg/kg) + (−)-DOI (0.3 mg/kg). VEH tests preceded each pharmacological challenge and were averaged across days for comparison.

**Effects of (−)-DOI on a Behavioral Economics Schedule of Cocaine IVSA.** Freely fed rats (n = 28) were trained on cocaine IVSA (0.75 mg/kg/inf) in 180-min sessions to allow for optimal conditions for the transition to the threshold procedure in which the first dose available is 0.75 mg/kg/inf (Oleson and Roberts 2009; Sun et al. 2021; Yates et al. 2019). Rats were initially trained on an FR1 schedule of cocaine IVSA until meeting a criterion with < 10% variation in the number of infusions received over three consecutive sessions. After demonstration of stability on an FR1 schedule, rats progressed to an FR3 schedule. After stable intake was achieved across a minimum of three FR3 training sessions, rats began daily sessions on the threshold procedure for a minimum of seven training days (Oleson and Roberts 2009; Sun et al. 2021; Yates et al. 2019). Throughout each daily session, rats earned progressively smaller doses of cocaine on an FR3 schedule. During the initial two 10-min time blocks, rats received 0.75 mg/kg/inf, however the first 10-min time block was excluded from data analysis as rats often “load up” consumption at the beginning of the session as the inclusion of the initial load-up phase tends to overestimate Q_0_ (Bentzley et al. 2013; McQueney and Garcia 2024; Oleson et al. 2011; Powell et al. 2020). Cocaine consumption recorded during the second time block was the first interval to be included in the generation of the demand curve. Following the first two time blocks, the duration of infusion decreased for the ten remaining time blocks which lasted 10-min each by 0.25 log per block for a total of 12 blocks (infusion duration of 6 s, 3.37 s, 1.89 s, 1.06 s, 0.59 s, 0.33 s, 0.19 s, 0.11 s, 0.06 s, 0.03 s, 0.02 s), effectively decreasing the dose of drug earned per infusion from 0.75 mg/kg/inf to 0.003 mg/kg/inf (Oleson and Roberts 2009; Sun et al. 2021; Yates et al. 2019). As a result, the price per unit (1 mg/kg) of cocaine increased from four responses to 1,000 responses by the final block of each session. Demand curves were fitted to the data for individual subjects in each session using an exponentiated demand equation (Hursh and Silberberg 2008; Koffarnus et al. 2015).

Pharmacological testing was initiated after stable performance was determined for each individual rat as less than 25% variability in demand intensity (**Q**_**0**_) and demand elasticity (alpha; **α**), and less than 10% variability in total intake across three consecutive sessions (Bentzley et al. 2013; Zimmer et al. 2012). Rats that failed to meet acquisition/maintenance criteria or lost catheter patency and were excluded from subsequent analyses (n = 17). Rats were tested in a pseudorandomized order with a dose–response curve of (−)-DOI (0.03, 0.1, 0.3 mg/kg), followed by combination tests with M100907 (0.01 mg/kg) + VEH and M100907 (0.01 mg/kg) + (−)-DOI (0.3 mg/kg).

### Statistical analyses

**Impact of (−)-DOI on Cocaine IVSA.** A within-subjects one-way analysis of variance (**ANOVA**) was employed to assess the impact of 5-HT_2A_R ligand treatment on cocaine infusions earned, active lever presses, inactive lever presses, and latency to first response. VEH tests preceding each pharmacological challenge were analyzed using a one-way ANOVA to ensure no differences were found across VEH tests which were averaged across days for statistical comparison. *A priori* comparisons versus VEH or (−)-DOI (0.3 mg/kg) for M100907 combination tests were defined prior to the start of experimentation and conducted with Sidak’s multiple comparisons analysis (Keppel 1973), when appropriate (Cunningham et al. 2011; Merritt et al. 2023; Neelakantan et al. 2017). All statistical analyses were conducted with GraphPad Prism (version 9.0.0) with an experiment-wise error rate set at α = 0.05. Investigators who performed drug administration and endpoint analyses were blinded to group assignments.

**Effects of (−)-DOI on a Behavioral Economics Schedule of Cocaine IVSA.** Demand curves for each subject were plotted with consumption (infusions * dose) as a function of FR requirement (price) and the data were fitted using the exponentiated demand model:${\mathbf{Q}=\mathbf{Q}}_{0}+{10}^{\mathbf{k}\left({\mathbf{e}}^{-{\varvec{\upalpha}}\left({\mathbf{Q}}_{0}\mathbf{C}\right)}-1\right)}$ in which $\mathbf{Q}$ represents consumption of cocaine, $\mathbf{C}$ represents the cost (the number of required lever presses for 1 mg/kg of cocaine), ${\mathbf{Q}}_{0}$ represents demand intensity (consumption at the lowest cost), ${\varvec{\upalpha}}$ represents demand elasticity (the decline in consumption with increased cost), and $\mathbf{k}$ is a scaling constant representing the range of consumption across the subjects (Hursh and Silberberg 2008). There is not consistent consensus in the literature on appropriate fitting of nonlinear demand curves, however some research investigations suggest an R^2^ value greater than 0.3 is sufficient (Bentzley et al. 2013; Murphy et al. 2009). In the present analyses, demand curves exhibited an R^2^ range of 0.84–0.99. The $\mathbf{k}$ constant was calculated each day and the highest calculated $\mathbf{k}$ value (3.72) was used for final data analysis. This calculation excluded behavior during the initial load-up bin based upon the fact that the “load-up phase” overestimates the true value of ${\mathbf{Q}}_{0}$ (Bentzley et al. 2013; McQueney and Garcia 2024; Oleson et al. 2011; Powell et al. 2020). ${\mathbf{P}}_{\mathbf{m}\mathbf{a}\mathbf{x}}$ is the price (in units of $\mathbf{C}$) at which the slope of the demand curve (i.e., price-point elasticity) is −1 and is inversely proportional to ${\varvec{\upalpha}}$(Espana et al. 2010; Lenoir and Ahmed 2008). ${\mathbf{P}}_{\mathbf{m}\mathbf{a}\mathbf{x}}$ is the price at which peak responding is achieved and corresponds with the peak response output (${\mathbf{O}}_{\mathbf{m}\mathbf{a}\mathbf{x}}$). In accordance with previous studies, the graphical values for ${\mathbf{P}}_{\mathbf{m}\mathbf{a}\mathbf{x}}$ were defined as the first price point that was followed consecutively by two price points with lower cocaine infusions (Espana et al. 2010; Lenoir and Ahmed 2008). ${\mathbf{O}}_{\mathbf{m}\mathbf{a}\mathbf{x}}$ was defined as the number of lever presses performed by the subjects at the ${\mathbf{P}}_{\mathbf{m}\mathbf{a}\mathbf{x}}$(Espana et al. 2010; Lenoir and Ahmed 2008). The essential value ($\mathbf{E}\mathbf{V}$) represents a scaled estimate that measures reinforcement strength-the degree to which a commodity is capable of maintaining behavior (Christensen et al. 2008). $\mathbf{E}\mathbf{V}$ was calculated as**:** ${\varvec{E}}{\varvec{V}}=\frac{1}{{\varvec{\upalpha}}\left({\mathbf{k}}^{1.5}\right)\mathbf{*}100}$(Hursh and Roma 2015; Kaplan and Reed 2014). Cocaine consumption was calculated from the number of infusions earned in each 10-min block multiplied by the dose administered. VEH tests preceded each pharmacological challenge. Given that α and Q_0_ were monitored throughout the entire study to ensure stability, these parameters were analyzed using a one-way ANOVA to ensure no differences were found across VEH tests. All parameters were averaged for statistical comparison. Differences in demand intensity (${\mathbf{Q}}_{0}$), demand elasticity (${\varvec{\upalpha}}$), total cocaine consumption, maximum expenditure (${\mathbf{O}}_{\mathbf{m}\mathbf{a}\mathbf{x}}$), essential value ($\mathbf{E}\mathbf{V}$), maximum price-point (${\mathbf{P}}_{\mathbf{m}\mathbf{a}\mathbf{x}}$), initial cocaine intake, and latency to first response were analyzed with a within-subjects one-way ANOVA. *A priori* comparisons versus VEH or (−)-DOI (0.3 mg/kg) for M100907 combination tests were defined prior to the start of experimentation and conducted with Sidak’s multiple comparisons analysis (Keppel 1973), when appropriate (Cunningham et al. 2011; Merritt et al. 2023; Neelakantan et al. 2017). All statistical analyses were conducted with GraphPad Prism (version 9.0.0) with an experiment-wise error rate set at α = 0.05. Investigators who performed drug administration and endpoint analyses were blinded to group assignments.

## Results

**Fig. 1:**
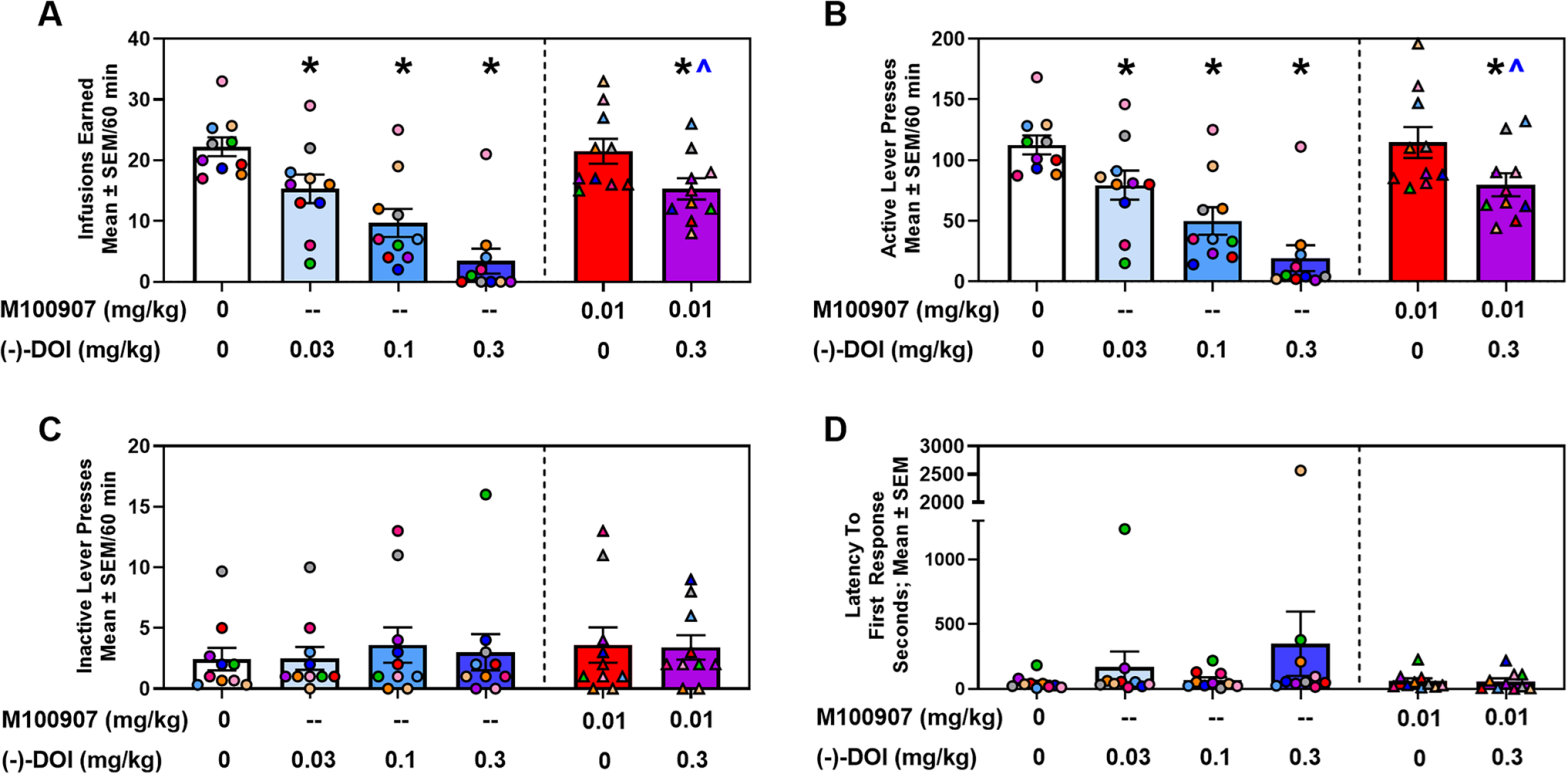
**Acute administration of (−)-DOI decreases cocaine (0.25 mg/kg/inf) self-administration (n = 10).** **A, Left Panel.** Pretreatment with (−)-DOI (0.03, 0.1, 0.3 mg/kg) decreases total cocaine infusions (0.25 mg/kg/infusion) (Mean ± SEM) relative to vehicle (*p < 0.05 vs. VEH). **A, Right Panel.** This effect is significantly reversed by the selective 5-HT_2A_R antagonist M100907 [^*p* < 0.05, M100907 (0.01 mg/kg) + (−)-DOI (0.3 mg/kg) vs. (−)-DOI (0.3 mg/kg)]. However, M100907 did not completely reverse the (−)-DOI-induced suppression of total cocaine infusions (*p < 0.05 vs. VEH). **B, Left Panel.** (−)-DOI pretreatment decreases active lever responses relative to control (*p < 0.05 vs. VEH). **B, Right Panel.** The effect of (−)-DOI on active lever presses is significantly reversed by M100907 [^*p* < 0.05, M100907 (0.01 mg/kg) + (−)-DOI (0.3 mg/kg) vs. (**−**)-DOI (0.3 mg/kg)]. However, M100907 did not completely reverse the (−)-DOI-induced suppression of total active lever presses (*p < 0.05 vs. VEH) **C, Left and Right Panel.** Neither (−)-DOI pretreatment nor M100907 alone or in combination with (−)-DOI have a significant effect on inactive lever responses (n.s.). **D, Left and Right Panel.** Latency to first active lever response was also unaffected (n.s.). Individual data points for each rat are represented by circles or triangles within each treatment

**(−)-DOI dose-dependently suppresses cocaine IVSA, an effect significantly blunted by 5-HT**_**2A**_**R antagonist pretreatment.** The efficacy of (−)-DOI (0.03–0.3 mg/kg; s.c.) was assessed in rats trained on cocaine IVSA (Fig. 1). Infusions earned during VEH tests were not different across days (F_4,40_ = 1.27, n.s.) and were averaged for statistical comparison. A main effect of (−)-DOI treatment was observed for infusions (F_5,45_ = 27.20, *p* < 0.05; Fig. 1A, left panel) and active lever presses (F_5,45_ = 23.27, *p* < 0.05; Fig. 1B, left panel), but not for inactive lever presses (F_5,45_ = 0.27, n.s.; Fig. 1C, left panel) or the latency to the first active lever response (F_5,45_ = 1.12, n.s.; Fig. 1D, left panel). *A priori *comparisons revealed that all doses of (−)-DOI (0.03, 0.1, 0.3 mg/kg) reduced the number of cocaine infusions (*p* < 0.05) and active lever responses versus VEH (*p* < 0.05).

M100907 (0.01 mg/kg; i.p.) alone did not alter cocaine intake (Fig. 1A**,** right panel) or other measures (Fig. 1B-1D**,** right panels) relative to VEH (n.s.), consistent with prior studies (Fletcher et al. 2002a; Murnane et al. 2013; Nic Dhonnchadha et al. 2009; Pockros et al. 2011). The combination of M100907 (0.01 mg/kg) plus (−)-DOI (0.3 mg/kg) resulted in significantly higher infusions earned (Fig. 1A, right panel) and active lever presses (Fig. 1B, right panel), relative to (−)-DOI (0.3 mg/kg) treatment alone (*p* < 0.05), suggesting a blunting of (−)-DOI-evoked suppression of cocaine intake. However, cocaine infusions earned (Fig. 1A, right panel) and active lever presses (Fig. 1B, right panel) remained significantly lower following the combination treatment relative to VEH-treated rats (*p* < 0.05). No significant differences were observed for inactive lever presses (Fig. 1C, right panel) or the latency to first response (Fig. 1D**,** right panel).

**Fig. 2:**
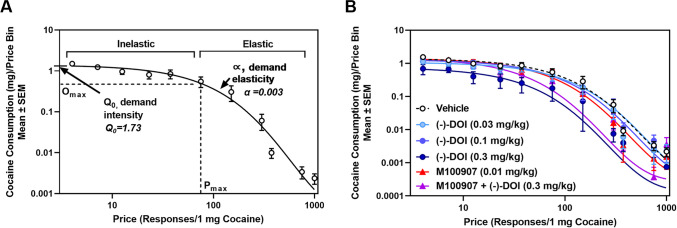
**Acute administration of (−)-DOI decreases cocaine demand (n = 11). A.** Example cocaine demand curve representing cocaine consumption as a function of price (lever presses/1 mg cocaine, in logarithmic units). Data points indicate cocaine consumption (mg) during the within-session threshold procedure from the cohort during a single session. α corresponds to the demand elasticity or the rate which cocaine consumption (mg) falls as the price increases (Hursh and Silberberg 2008). Q_0_ corresponds to the demand intensity, defined as the “hedonic setpoint” or cocaine consumption (mg) at the lowest price estimated by the demand curve (Hursh and Silberberg 2008). P_max_ corresponds to the price at which demand shifts from inelastic to elastic, as defined at the point at which the slope of the demand curve is −1 (Espana et al. 2010; Lenoir and Ahmed 2008). O_max_ corresponds to the cocaine dosage available at the P_max_ price point (Espana et al. 2010; Lenoir and Ahmed 2008). **B.** Acute (−)-DOI administration results in a leftward shift of the cocaine demand curve, indicating increased demand elasticity (decreased motivation). Mean data points for each treatment group are represented by circles or triangles within each treatment

**(−)-DOI suppresses cocaine IVSA in a behavioral economics schedule.** We examined the effects of (−)-DOI in a behavioral economics schedule of cocaine IVSA (Fig. 2). Following stable acquisition of IVSA, rats underwent a minimum of seven training days on the threshold procedure until stabilization criteria were met. Demand curves were plotted as the number or reinforcers earned (consumption, mg; in logarithmic units) as a function of FR requirement (price) using the exponential model of Hursh and Silberberg: ${\mathbf{l}\mathbf{o}\mathbf{g}\mathbf{Q}=\mathbf{l}\mathbf{o}\mathbf{g}\mathbf{Q}}_{0}+\mathbf{k}\left({\mathbf{e}}^{-{\varvec{\upalpha}}\left({\mathbf{Q}}_{0}\mathbf{C}\right)}-1\right)$ (Fig. 2A-2B) (Hursh and Silberberg 2008). An example curve is plotted to demonstrate cocaine consumption (mg) as a function of price (lever presses/1 mg cocaine) following vehicle administration (Fig. 2A). The impact of the (-)-DOI dose–response curve (0.03–0.3 mg/kg; s.c.) on demand curves is shown (Fig. 2B).

**Fig. 3:**
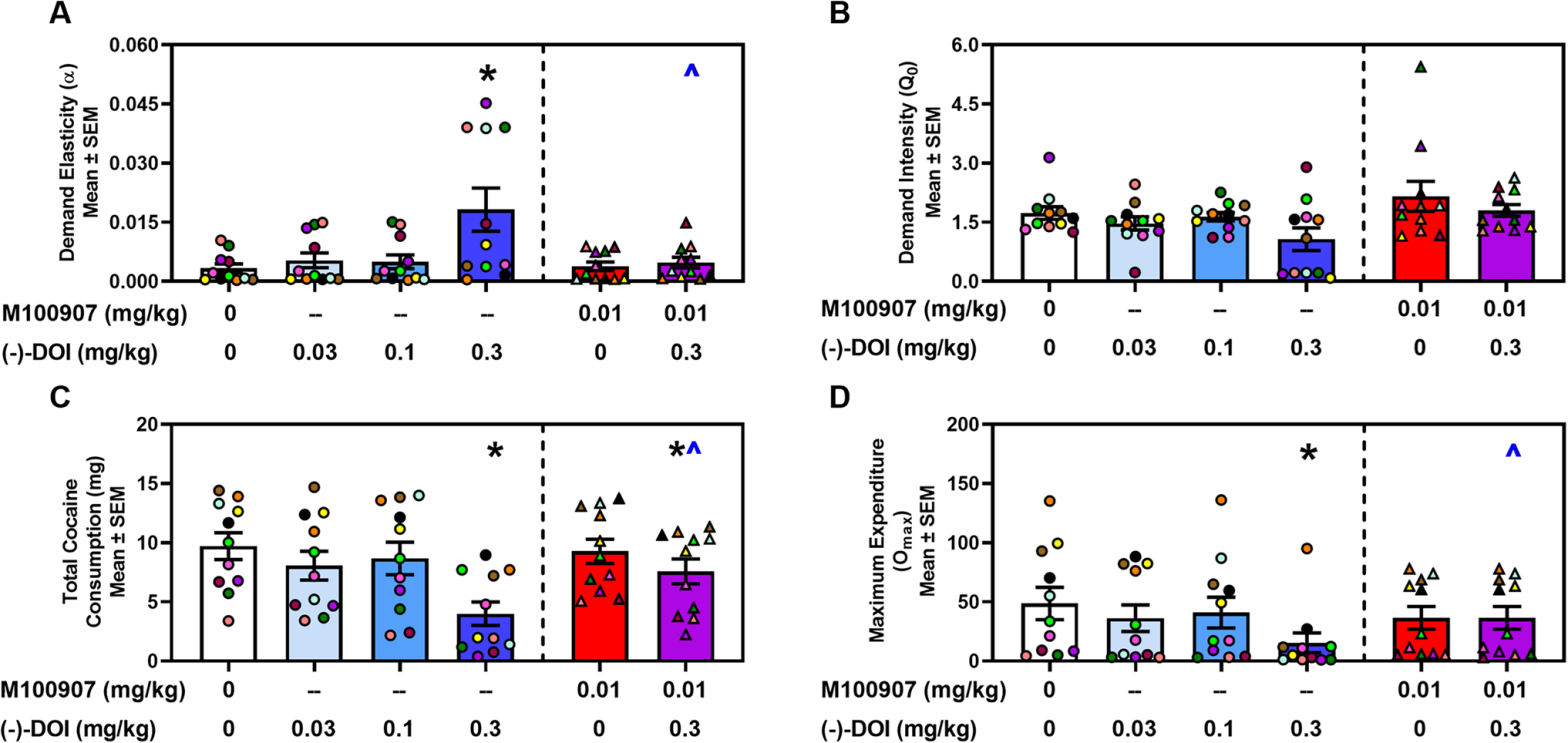
**Acute administration of (−)-DOI increases sensitivity to price changes and decreases cocaine consumption at null cost and total cocaine consumption during self-administration (n = 11). A, Left Panel. **(−)-DOI pretreatment significantly elevated α following acute (−)-DOI administration (0.3 mg/kg) (*p < 0.05 vs VEH). **A, Right Panel.** The effect of (−)-DOI on α was reversed by pretreatment with M100907 (0.01 mg/kg) [^*p* < 0.05, M100907 (0.01 mg/kg) + (−)-DOI (0.3 mg/kg) vs. (−)-DOI (0.3 mg/kg); n.s. vs VEH]. **B, Left Panel.** A main effect of (−)-DOI pretreatment on Q_0_ was identified (*p* = 0.05 vs VEH). **B, Right Panel.** M100907 (0.01 mg/kg) alone or in combination with (−)-DOI (0.3 mg/kg) had no effect on Q_0_ (n.s.). **C, Left Panel**. (−)-DOI pretreatment (0.3 mg/kg) significantly decreased total cocaine consumption (mg) during the self-administration session (*p < 0.05). **C, Right Panel.** This effect was significantly reversed with M100907 pretreatment [^*p* < 0.05, M100907 (0.01 mg/kg) + (−)-DOI (0.3 mg/kg) vs. (−)-DOI (0.3 mg/kg); n.s. vs VEH]. **D, Left Panel.** A main effect of (−)-DOI pretreatment was found on O_max_ (F_5,50_ = 3.662, *p* < 0.05). Pretreatment with (−)-DOI (0.3 mg/kg) significantly decreased O_max_ (*p < 0.05). **D, Right Panel.** The effect of (−)-DOI on O_max_ was prevented by pretreatment with M100907 [^*p* < 0.05, M100907 (0.01 mg/kg) + (−)-DOI (0.3 mg/kg) vs. (−)-DOI (0.3 mg/kg); n.s. vs VEH]. Individual data points for each rat are represented by circles or triangles within each treatment

The parameters extrapolated from cocaine IVSA demand curves are presented in Fig. 3A-3D and Table 1. Demand behavior during VEH tests was not different across days (α: F_4,40_ = 0.43, n.s.; Q_0_: F_4,40_ = 0.72, n.s.) and all parameters were averaged for statistical comparison. A main effect of (−)-DOI treatment was observed for α (F_5,50_ = 8.038, *p* < 0.05; Fig. 3A**,** left panel), Q_0_ (F_5,50_ = 2.353, *p* = 0.05; Fig. 3B**,** left panel), total cocaine intake (mg) during the session (F_5,50_ = 15.41, *p* < 0.05; Fig. 3C**,** left panel), O_max_ (F_5,50_ = 4.178, *p* < 0.05; Fig. 3D**,** left panel), EV (F_5,50_ = 4.469, *p* < 0.05; Table 1), P_max_ (F_5,50_ = 2.831, *p* < 0.05; Table 1), and ‘load up period’ intake (F_5,50_ = 11.75,* p* < 0.05; Table 1), referring to the first ten minutes of the session (Bentzley et al. 2013; Oleson et al. 2011; Powell et al. 2020). *A priori* comparisons revealed that the dose of 0.3 mg/kg (−)-DOI significantly increased α (*p* < 0.05; Fig. 3A**,** left panel) and decreased total cocaine intake during the session (*p* < 0.05; Fig. 3C**,** left panel), O_max_ (*p* < 0.05; Fig. 3D**,** left panel), EV (*p* < 0.05; Table 1), P_max_ (*p* < 0.05; Table 1), and cocaine consumption in the first ten minutes (*p* < 0.05; Table 1) compared to saline treatment. A main effect of treatment with (-)-DOI on Q_0_ was observed, however the multiple comparisons analysis failed to detect significance differences between treatment groups. There was no main effect of treatment on latency to first active lever presses (F_5,50_ = 1.678, n.s.; Table 1).

**Table 1:** Effects of acute (−)-DOI on measures of cocaine demand (Mean ± SEM)

Treatment	EV	P_max_	Load Up Period (mg)	Latency (sec)
VEH	1.48 ± 0.42	85.98 ± 24.02	3.43 ± 0.24	23.52 ± 3.01
(−)-DOI 0.03 mb/kg	1.11 ± 0.35	70.85 ± 18.85	2.93 ± 0.24	19.91 ± 3.28
(−)-DOI 0.1 mg/kg	1.25 ± 0.40	70.80 ± 21.54	3.27 ± 0.31	34.82 ± 10.97
(−)-DOI 0.3 mg/kg	0.50 ± 0.25*	48.16 ± 18.89*	1.57 ± 0.39*	117.60 ± 69.60
VEH + M100907 0.01 mg/kg	1.11 ± 0.29	62.87 ± 17.87	3.48 ± 0.16	45.82 ± 8.01
(−)-DOI 0.3 + M100907 0.01 mg/kg	0.73 ± 0.22*	44.02 ± 14.48*	2.93 ± 0.24**^**	27.45 ± 3.51

^*^ *p* < 0.05 vs. VEH

^ *p* < 0.05 vs. (−)-DOI (0.3 mg/kg)

M100907 (0.01 mg/kg; i.p.) treatment alone did not alter extrapolated parameters of the cocaine demand curve relative to VEH (n.s.; Fig. 3A and D, right panels). The combination of M100907 (0.01 mg/kg) plus (−)-DOI (0.3 mg/kg) significantly decreased α (*p* < 0.05; Fig. 3A**,** right panel) and increased total cocaine consumption (*p* < 0.05; Fig. 3C**,** right panel), O_max_ (*p* < 0.05; Fig. 3D**,** right panel), and consumption during the first 10 min (*p* < 0.05; Table 1), relative to (−)-DOI (0.3 mg/kg). However, M100907 did not prevent the impact of (−)-DOI on EV or P_max_ (n.s.; Table 1).

## Discussion

Our study found that (−)-DOI (0.03–0.3 mg/kg) dose-dependently reduced intake on an FR5 schedule of cocaine IVSA and that the highest dose of (−)-DOI (0.3 mg/kg) left shifted the demand curve and evoked greater sensitivity to price increases in the behavioral economics paradigm. Pretreatment with M100907 (0.01 mg/kg) did not completely reverse (-)-DOI-induced suppression of cocaine intake to baseline levels on the FR5 schedule, although pilot studies indicate that M100907 (0.1 mg/kg) fully reversed (−)-DOI-induced (0.3 mg/kg) inhibition of cocaine IVSA (0.25 mg/kg/inf) in male rats [F_2,20_ = 131.8, *p* < 0.05 versus 0.3 mg/kg (−)-DOI]. In addition, M100907 (0.01 mg/kg) reversed (−)-DOI-evoked (0.3 mg/kg) decreases in α, total cocaine consumption, O_max_, and consumption during the first ten minutes in the behavioral economics paradigm. Thus, (−)-DOI ‘devalued’ cocaine reward in a 5-HT_2A_R-dependent manner. In sum, we uncovered the first evidence that acute pretreatment with the psychedelic (−)-DOI decreases the reinforcing properties of cocaine and persistent intake under high-effort conditions.

The within-session threshold procedure is an advanced IVSA model designed to evaluate reinforcement as well as motivation (Bentzley et al. 2014; Galuska et al. 2011; James et al. 2019). The resultant leftward shift in the cocaine demand curve following (−)-DOI administration and increase in demand elasticity (α), indicated enhanced price sensitivity or a devaluation of cocaine reinforcement (Hursh 1993; Hursh and Silberberg 2008; Hursh and Winger 1995). A main effect of (−)-DOI treatment was observed (*p* = 0.05) on demand intensity (Q_0_) which is defined as cocaine consumption at the lowest available price; however, preplanned comparisons lacked significance. Visual inspection of the data reveals a notable reduction in Q_0_ in a subset of animals, suggesting a change in the median consumption. Indeed, the median Q_0_ value following vehicle treatment was 1.6, relative to 1.1 following the 0.3 mg/kg dose of (−)-DOI. This shift in distribution suggests that (−)-DOI lowers consumption at near free prices in a subset of individuals. As the within-session threshold procedure begins with the highest available dose of cocaine (0.75 mg/kg/inf) to mimic a free market system (Oleson and Roberts 2009; Sun et al. 2021; Yates et al. 2019), this lack of statistical significance may indicate that, while (−)-DOI devalues cocaine under conditions in which the ‘price’ is higher, (−)-DOI may be less efficacious in some individuals when cocaine is relatively ‘free’ to self-administer (Oleson and Roberts 2009; Sun et al. 2021; Yates et al. 2019). (−)-DOI (0.3 mg/kg) also significantly suppressed intake during the load-up period, relative to vehicle-treated rats. This decrease may be interpreted as increased or decreased sensitivity to cocaine. However, when interpreted with α, we are confident that (−)-DOI is not enhancing the reinforcing strength of cocaine. (−)-DOI also decreased the theoretical EV, P_max_, and O_max_, indicating a devaluation in cocaine reward (EV) leading to decreased motivation to continue drug taking when more effort is required (P_max,_ O_max_) to obtain the desired dosage of cocaine. Notably, the changes in α, total cocaine consumption, O_max_, and consumption during the load up period resulting from acute (−)-DOI pretreatment were blunted by pretreatment with the selective 5-HT_2A_R antagonist M100907. Observations from this study suggest that (−)-DOI reduces motivation for cocaine through activation of 5-HT_2A_R. As the behavioral economics assay is predictive of additional translational aspects of CUD (Bentzley et al. 2014; Strickland et al. 2016), a psychedelic may evoke enduring beneficial effects to decrease cocaine intake and cocaine-seeking during abstinence in addition to the acute suppressive effects on cocaine-taking demonstrated here.

Cocaine exposure induces complex changes within the serotonergic system (for review) (Cunningham et al. 2020). For example, altered 5-HT_2A_R expression and/or function are reported during withdrawal from repeated cocaine exposure in rodents (Baumann and Rothman 1996; Carrasco et al. 2004, 2003; Darmani et al. 1992; Huang et al. 2009) and increased frontal cortical 5-HT_2A_R levels were observed following extended cocaine IVSA in primates (Sawyer et al. 2012). One caveat to consider is that large doses of IVSA cocaine are available in the early time bins of sessions in the behavioral economics procedure (Oleson and Roberts 2009; Sun et al. 2021; Yates et al. 2019). In the present studies, rats consumed an average of 2.07 ± 0.14 mg of cocaine (mean ± SEM) in the entire 60-min FR paradigm relative to an average of 8.78 ± 2.93 mg of cocaine in the first 60 min of the behavioral economics schedule, constituting a 4.25-fold higher cocaine intake relative to FR IVSA animals. The magnitude of cocaine intake exposure may result in differential 5-HT_2A_R functionality to explain the discrepancy between effective doses of (−)-DOI (0.03–0.3 mg/kg) in standard IVSA paradigm relative to the behavioral economics procedure (0.3 mg/kg). Future studies are required to empirically examine this possibility.

Attentional processing is altered in rats exposed to cocaine self-administration (Vazquez et al. 2020) as well as human cocaine abusers (Tomasi et al. 2007). 5-HT_2A_R activation by a psychedelic also impacts these processes in humans (Kometer et al. 2013) and rats (Zhang et al. 2017). Evidence suggests that higher doses of DOI than those used in the current study may affect temporal perception processing (Hampson et al. 2010; Michaiel et al. 2019) and auditory or visual prepulse inhibition in rodents (Padich et al. 1996). The dose range of (−)-DOI (0.03–0.3 mg/kg) in the present studies does not overtly modify spontaneous behaviors (Bishop et al. 2004; Filip et al. 2004) nor operant responding on inactive levers or the latency to first active lever response in cocaine IVSA (current results), evidence in support of inability of (−)-DOI to impact performance of the operant cocaine IVSA assay. Future research is necessary to examine the effects of the tested dose range of (−)-DOI on sensory and perceptual processes within the context of cocaine self-administration.

(-)-DOI was employed as an investigational psychedelic in the current studies for its higher 5-HT_2A_R selectivity over other 5-HT receptors (Barnes et al. 2021; Kehne et al. 1996), however, formal studies of (−)-DOI in healthy human volunteers nor clinical patients have not been conducted. Psilocybin in tandem with Motivational Enhancement therapy reduced heavy alcohol intake maintained at follow-up to 36 weeks after one or two supervised sessions in participants diagnosed with alcohol dependence (Bogenschutz et al. 2015). An open-label pilot study of psilocybin in nicotine-dependent smokers in the context of psychosocial support demonstrated that 80% of participants achieved abstinence at 6-month follow-up which exceeds other nicotine cessation treatments (Johnson et al. 2014). Psychedelics are currently highlighted as potential therapeutics for CUD (Jones and Nock 2022; Yaden et al. 2024) and a Phase 2 study of psilocybin for CUD was recently completed (NCT02037126) and an early Phase 1 safety study in CUD participants is soon to start (NCT06102434). Investigations of 5-HT_2A_R-acting psychedelics (e.g., psilocybin) in preclinical analyses of cocaine intake and relapse vulnerability will be valuable as prelude to future clinical trials. Moreover, new medicinal chemistry initiatives are underway to identify novel selective 5-HT_2A_R agonists with distinct pharmacological profiles (Cameron et al. 2021; Cunningham et al. 2023; Lewis et al. 2023). The recent discovery of novel positive allosteric modulators that enhance 5-HT_2_R signaling without directly binding the orthosteric site provides a new perspective to selectively target the 5-HT_2A_R for further investigation, engaging 5-HT_2A_R-mediated biological mechanisms distinct from serotonergic psychedelics (Chen et al. 2023; Wild et al. 2019; Wold et al. 2020). Therefore, the data reported herein represent the first studies suggesting promising efficacy of a 5-HT_2A_R agonist to reduce cocaine intake and continued innovation in medicinal chemistry and drug development will allow for future studies to selectively target the 5-HT_2A_R to modulate cocaine reward.

Our studies profiled (−)-DOI in male rats and additional research is required to determine (−)-DOI efficacy in female rats which respond differently to cocaine based on hormonal status (Doncheck et al. 2021; Sell et al. 2005, 2000, 2002) and distinct facets of cocaine self-administration versus male rats (Algallal et al. 2020; Doncheck et al. 2021; Lynch and Carroll 1999). While the 5-HT_2A_R agonist-mediated head-twitch response in mice is comparable between sexes (Darmani et al. 1996), it cannot be assumed the behavioral sensitivity of (−)-DOI to reduce drug intake will be the same following chronic cocaine exposure. In conclusion, we demonstrate the efficacy of (−)-DOI to decrease cocaine intake and demand in a 5-HT_2A_R-mediated manner. Although the reported studies demonstrate 5-HT_2A_R as an important target for therapeutic effects of (−)-DOI, the precise mechanisms through which (−)-DOI acts to reduce cocaine intake and motivation have yet to be reported in detail. Thus, future research is needed to delineate the neurobiological and molecular mechanisms through which (−)-DOI exerts its effects. As serotonergic psychedelics continue to emerge as potential therapeutics for SUDs, further preclinical investigations are warranted to elucidate the greater role of 5-HT_2A_R signaling in drug reward. Given that economic demand for cocaine predicts an addiction-like behavioral phenotype and pharmacotherapeutic efficacy in rodents and humans (Bentzley et al. 2014; James et al. 2019; McNally et al. 2023), continued investigation into the unique aspects of psychedelic and non-psychedelic 5-HT_2A_R agonists in this model will provide important evidence concerning future opportunities for therapeutic intervention in CUD.

## Data Availability

The authors confirm that the data supporting the findings of this study are available within the article.
